# Public reporting of PCI operator outcomes

**DOI:** 10.18632/aging.102624

**Published:** 2019-12-17

**Authors:** Matthew D. Kelham, Andrew Wragg, Daniel A. Jones

**Affiliations:** 1 Department of Cardiology, Barts Heart Centre, Barts Health NHS Trust, West Smithfield, London EC1A 7BE, UK

**Keywords:** outcomes, percutaneous coronary intervention

Public reporting of healthcare system and provider outcomes is believed by its proponents to facilitate transparency, incentivise high quality care and allow patients to make informed decisions in choosing healthcare providers. The existing evidence base for public reporting is predominantly derived from studies of North American patients who have undergone cardiac procedures, with systematic reviews and meta-analyses suggesting the impact of public reporting on clinical outcomes is mostly positive and may reduce mortality, although with a high degree of heterogeneity between studies that limits this interpretation [1,2].

However, despite increasing enthusiasm for public reporting internationally, it remains a contentious issue. This is particularly the case with respect to public reporting of individual percutaneous coronary intervention (PCI) operator outcomes, with its critics highlighting both whether one is able to accurately risk adjust to make fair assessments but also that public reporting may lead to risk adverse behaviour by physicians [3] The latter criticism is supported by both indirect and direct evidence that public reporting of operator outcome influences interventional cardiologists’ decision to proceed to PCI. One study from the United States highlights, in a sample of over 80,000 patients admitted with acute myocardial infarction, that in states with public reporting the rates of PCI are lower than in those states without public reporting [4]. Of concern was that this effect was most pronounced in the patient groups (cardiogenic shock, arrest) with potentially the most to benefit from urgent revascularisation [4]. After patients with cardiogenic shock were excluded from public reporting in New York there was a significant increase in the rates of PCI in shock with a corresponding decrease in in-hospital mortality [5]. Furthermore, in a survey of interventional cardiologists from New York and Massachusetts almost two-thirds of respondents admitted avoiding PCI on at least two occasions because of concerns that a bad outcome would negatively impact their publicly reported outcomes [6]. There is also concern that the practice of public reporting of PCI outcomes imposes a significant financial and administrative burden on interventional cardiologists and their hospitals [7].

As highlighted above, previous studies assessing the impact of public reporting of operator outcomes related to PCI are overwhelmingly derived from North America [2]. In Europe, there is limited data with only the UK adopting the public reporting of individual PCI operator outcomes in 2012 by the British Cardiovascular Intervention Society (BCIS). The impact of this introduction has been recently assessed by this group, in a cohort study of over 120,000 consecutive PCI patients in London between 2005 and 2015 [8]. Over time it was clear that the risk factor profile of patients was changing. Treated patients were older, had more complex medical problems (higher incidence of Type 2 diabetes, chronic kidney disease and previous revascularisation), were more likely to be in cardiogenic shock or post out of hospital cardiac arrest and the dominant indication became acute coronary syndrome rather than elective PCI. Despite this increase in patient risk factor profiles a lower incidence of in hospital major adverse cardiovascular and cerebrovascular events (MACCE) were noted over time and post the introduction of public reporting. 30-day mortality rates were examined using interrupted time series analysis to compare the periods before and after introduction of public reporting. We demonstrated that in the time period prior to public reporting 30-day mortality rates increased significantly (reflective of an increasing patient risk profile), however the introduction of public reporting was associated with a decrease in 30-day mortality of 35%. No evidence of patient selection bias was seen to suggest risk adverse behaviour.

This study suggests that, in the UK, the introduction of public reporting of PCI operator outcomes has been associated with an improvement in outcomes without evidence of the risk adverse behaviour previously demonstrated in North American studies. Whilst this contrast may reflect methodological differences between the studies the question should be raised as to why there would be a variance in response to reporting between physicians on either side of the Atlantic. The distinction may relate to the difference in the structure and funding of healthcare, whereby UK physicians working in the NHS feel less compelled to change their behaviour in response to public reporting than operators working in an environment where patients may have more choice regarding which operator they consult. However, as noted elsewhere, patients requiring urgent PCI are unlikely to have the opportunity for much say regarding their operator and there is no great evidence to suggest patients use public reports that are available [3]. Additionally, further work is needed on the quality improvement measures used with better metrics such as patient reported outcome measures (quality of life, angina scores) needed.

The public reporting of PCI operator outcomes is being increasingly adopted internationally. Whilst there are ongoing concerns regarding whether public reporting leads to risk adverse behaviour, particularly in patients with the most to gain from PCI, there is a continuing need for careful interrogation on the impact of reporting. Whether the impact of public reporting varies dependent on the healthcare system in which physicians operate, and which metrics should be used, are of interest and should be considered in future studies.
